# Supplementary material for: Comparative multiomics analysis of cell physiological state after culture in a basket bioreactor
Source: Sci Rep. 2022 Nov 23;12:20161. doi: 10.1038/s41598-022-24687-4 (PMC9686226; doi:10.1038/s41598-022-24687-4)
Supplement: Supplementary file 1 — Supplementary Information 1. [file 41598_2022_24687_MOESM1_ESM.zip › raw data/Transcriptomic raw data/6.Enrichment/2.KEGG/TE1vsTC1/GSEA/METABOLIC_PATHWAYS(CSAB01100).html]

Details for gene set METABOLIC\_PATHWAYS(CSAB01100)[GSEA]

|  || Dataset | readcount.sample |
| Phenotype | sample.cls |
| Upregulated in class | TE1 |
| GeneSet | METABOLIC\_PATHWAYS(CSAB01100) |
| Enrichment Score (ES) | 0.57240033 |
| Normalized Enrichment Score (NES) | 1.2452099 |
| Nominal p-value | 0.0 |
| FDR q-value | 0.3752867 |
| FWER p-Value | 0.816 |
Table: GSEA Results Summary

  

Fig 1: Enrichment plot: METABOLIC\_PATHWAYS(CSAB01100)      
 Profile of the Running ES Score & Positions of GeneSet Members on the Rank Ordered List

  

| PROBE | DESCRIPTION (from dataset) | GENE SYMBOL | GENE\_TITLE | RANK IN GENE LIST | RANK METRIC SCORE | RUNNING ES | CORE ENRICHMENT || 1 | novel.572 | - |  |  | 628 | 10.870 | 0.5724 | Yes |
| 2 | novel.518 | - |  |  | 8940 | 1.089 | 0.2614 | No |
| 3 | novel.479 | - |  |  | 9582 | 1.049 | 0.2907 | No |
| 4 | novel.517 | - |  |  | 11208 | 0.944 | 0.2703 | No |
| 5 | novel.514 | - |  |  | 14470 | 0.741 | 0.1656 | No |
| 6 | novel.471 | - |  |  | 15225 | 0.693 | 0.1702 | No |
| 7 | novel.529 | - |  |  | 15745 | 0.658 | 0.1834 | No |
| 8 | novel.530 | - |  |  | 16765 | 0.586 | 0.1703 | No |
| 9 | novel.574 | - |  |  | 18341 | 0.456 | 0.1251 | No |
| 10 | novel.516 | - |  |  | 18769 | 0.414 | 0.1289 | No |
| 11 | novel.266 | - |  |  | 18854 | 0.407 | 0.1476 | No |
| 12 | novel.503 | - |  |  | 20481 | 0.195 | 0.0858 | No |
Table: GSEA details [plain text format]

  

Fig 2: METABOLIC\_PATHWAYS(CSAB01100)      
 Blue-Pink O' Gram in the Space of the Analyzed GeneSet

  

Fig 3: METABOLIC\_PATHWAYS(CSAB01100): Random ES distribution      
 Gene set null distribution of ES for **METABOLIC\_PATHWAYS(CSAB01100)**
